# Dietary Modification Patterns and Interventions Among Weight Loss Seekers in Tamil Nadu, South India: A Cross-Sectional Analysis of Intermittent Fasting and Alternative Dietary Approaches

**DOI:** 10.7759/cureus.76647

**Published:** 2024-12-30

**Authors:** Tamilarasan Muniyapillai, Neethu George, Rock Britto Dharmaraj, Akaash Parthasarathi, Naveen Panneerselvan, Aravindhan Thirumalraj, A Jeganish, Karthikeyan Kulothungan

**Affiliations:** 1 Community Medicine, Dhanalakshmi Srinivasan Medical College and Hospital, Siruvachur, IND; 2 Emergency Medicine, Christian Medical College, Vellore, IND; 3 Geriatrics, All India Institute of Medical Sciences, New Delhi, IND; 4 Internal Medicine, Vijaya Hospital, Chennai, IND; 5 Community Medicine, All India Institute of Medical Sciences, Mangalagiri, Guntur, IND

**Keywords:** dietary habits, dietary modifications, dietetics, intermittent fasting, weight loss diets, weight loss interventions

## Abstract

Background

The escalating global obesity epidemic requires comprehensive investigations for effective weight management strategies. Understanding the patterns, barriers, and facilitators of dietary interventions is crucial for developing effective weight management protocols. This research aims to assess dietary modification interventions among weight loss subjects in Tamilnadu, South India. Specific objectives included evaluating various weight loss interventions, analyzing dietary patterns adopted by subjects, and examining characteristics, barriers, and facilitators associated with specific dietary modification approaches.

Methodology

A cross-sectional study was conducted among 432 participants from Tamilnadu, South India. The research employed a comprehensive data collection approach, gathering information on demographic characteristics, anthropometric measurements, dietary modification patterns, and intervention outcomes. Participants were categorized based on their chosen dietary interventions, particularly distinguishing between intermittent fasting and alternative dietary approaches. The study systematically evaluated various parameters, including regular adherence, physiological effects, psychological impacts, and barriers to maintenance. Statistical analysis utilized chi-square and Fischer's exact tests for categorical variables, while independent t-tests were employed for continuous variables.

Results

This study encompassed 432 participants, with demographically diverse participants characterized by a predominance of urban residents (295, 68.3%), highly educated individuals (383, 88.6%), and students (190, 44%). The mean age was 27.93 years, with a mean body mass index of 25.21 kg/m^2^. Regarding dietary intervention objectives, the study revealed that 289 (66.9%) were not engaged in any healthcare intervention, while 102 (23.6%) pursued intermittent fasting and 41 (9.5%) adopted alternative dietary strategies such as Paleo and ketogenic diets. Among participants implementing dietary modifications, the mean intervention duration was 5.21 months, ranging from half a month to 60 months. Intermittent fasting participants exhibited statistically significant characteristics, including a younger mean age (27.09 years) compared with alternative diet groups (37.37 years). Notably, 73 (71.6%) reported significant weight loss, with 69 (67.6%) experiencing weight regain during non-adherence. Psychological and physiological benefits were prominently observed, with 73 (71.6%) reporting mood improvements, 71 (69.6%) experiencing enhanced concentration, and 72 (70.6%) noting improved bowel habits. Barriers to dietary modifications included timing challenges (50, 49%), family mealtime conflicts (43, 42.2%), and work schedule interruptions (39, 38.2%).

Conclusion

The research provides comprehensive insights into dietary modification patterns among young Indian adults, highlighting intermittent fasting's potential as an effective weight management strategy. The findings underscore the complex interplay between dietary choices, individual characteristics, and holistic health outcomes. While demonstrating promising weight loss and cognitive benefits, the study emphasizes the necessity of personalized, context-sensitive nutritional interventions. These insights contribute significantly to understanding dietary modification dynamics and inform the development of more effective, tailored weight management strategies.

## Introduction

The global prevalence of obesity represents a significant public health challenge characterized by complex interactions between dietary behaviors, lifestyle modifications, and physiological mechanisms [1,2]. Emerging research indicates that targeted dietary interventions play a crucial role in weight management strategies, with multiple approaches demonstrating potential efficacy in addressing this pervasive health concern [3,4].

Contemporary nutritional science has increasingly focused on understanding diverse dietary modifications and their impact on weight loss outcomes [5,6]. Intermittent fasting, ketogenic diets, and other specialized dietary approaches have gained substantial attention as potential alternatives to traditional continuous energy restriction methods. These contemporary strategies reflect a paradigm shift in weight management, transitioning from simplistic caloric reduction towards more intricate metabolic interventions that target fundamental physiological processes [7,8].

Epidemiological data suggests that obesity is not merely a metabolic disorder, but a complex condition influenced by multiple factors, including genetic predisposition, environmental contexts, and individual behavioral patterns [2,8,9]. The heterogeneity of weight loss interventions underscores the necessity for comprehensive research exploring various dietary modification strategies and their differential impacts on metabolic health [3,4,10].

Recent systematic reviews have highlighted the importance of understanding barriers and facilitators associated with dietary modifications [11-13]. These investigations reveal that successful weight loss interventions extend beyond nutritional recommendations, encompassing psychological, social, and environmental dimensions [14-16]. The complexity of adherence to dietary regimens necessitates a multifaceted approach that considers individual variability in response to nutritional interventions [17,18].

Intermittent fasting and alternative dietary approaches have emerged as promising strategies for weight management, demonstrating potential metabolic benefits beyond traditional weight loss methodologies [19-21]. Clinical studies have explored their efficacy in weight reduction, metabolic regulation, and potential long-term health outcomes [22-24]. However, the variability in individual responses and the potential systemic implications require continued rigorous scientific investigation [7,25].

The current study aims to assess dietary modification patterns, focusing on intermittent fasting and alternative dietary interventions. By examining the characteristics, barriers, and facilitators associated with these dietary approaches, we seek to contribute valuable insights into the complex landscape of weight management strategies. Understanding the nuanced interactions between dietary interventions and individual physiological responses can potentially inform more personalized and effective weight loss approaches.

## Materials and methods

Study design and study setting

A cross-sectional analytical study was conducted to assess dietary modification patterns among weight loss seekers in Tamil Nadu, South India. The study was carried out at Dhanalakshmi Srinivasan Medical College, which serves as a tertiary healthcare center catering to both urban and rural populations in Tamilnadu, a southern state in India.

Study period

The study was conducted over a six-month period from October 2023 to March 2024, allowing for adequate sample collection and comprehensive data analysis.

Ethics committee approval

The study protocol received approval from the Institutional Ethics Committee of Dhanalakshmi Srinivasan Medical College (approval number: IECHS/IRCHS/No.401-B). Digital informed consent was obtained from all participants before data collection, ensuring adherence to ethical research principles.

Inclusion criteria

The study included individuals aged 18 years and above, residing in Tamil Nadu, who were actively seeking weight loss interventions. Both males and females were included, regardless of their current BMI status or previous weight loss attempts.

Exclusion criteria

Participants were excluded if they had any severe chronic illnesses affecting dietary patterns or were pregnant or lactating. Those following physician-prescribed therapeutic diets for specific medical conditions were also excluded.

Sample size estimation

The sample size was calculated using Cochran's formula: n = Z^2^ × p × (1-p) / d^2^, where Z = 1.96 (95% confidence level), p = 0.33 (prevalence of dietary modifications from previous study), and d = 0.05 (margin of error). The prevalence of dietary modifications (p) was determined from previous research by Welton et al. (2020), who reported a 33% prevalence of intermittent fasting adoption among weight loss seekers in their systematic review of 27 studies involving adult populations [20]. The calculated sample size was 340. Accounting for a 20% non-response rate typically observed in online surveys (340 + 68 = 408), the final sample size was rounded to 432 participants to ensure adequate statistical power and compensate for potential incomplete responses.

Sampling method

A convenience sampling technique was employed, utilizing an online survey platform (Google Forms) distributed through social networks within Tamil Nadu. This method was chosen to ensure broad geographical coverage and accessibility during the study period, similar to approaches used in contemporary dietary research.

Data collection procedure

A comprehensive structured questionnaire was developed through a rigorous literature review and expert consultation. The questionnaire underwent a two-phase validation process: initial content validation through review by a panel of three experts (two nutritionists and one community medicine specialist), followed by construct validation through a pilot study involving 30 participants (not included in the final analysis) (see Appendix). Minor modifications were implemented based on pilot study feedback to enhance clarity and user understanding.

The finalized questionnaire encompassed comprehensive sections to capture the full spectrum of participant characteristics and intervention outcomes. The demographic profile section collected data on age, gender, residential status, educational attainment, and occupational categories. Anthropometric measurements included self-reported height (cm), weight (kg), and calculated body mass index (kg/m^2^). The dietary intervention section assessed the type of modification (intermittent fasting, alternative diets including Paleo and ketogenic), duration of the intervention (in months), and implementation patterns. Participants' lifestyle modifications were evaluated through questions about physical activity engagement, mind-body interventions (yoga, meditation), and other health-seeking behaviors. The questionnaire incorporated a detailed assessment of intervention outcomes, including weight loss achievement, systemic effects, psychological impacts (mood alterations, memory concerns), sleep duration changes, and weight regain patterns during non-adherence periods. Implementation challenges were assessed through questions about timing constraints, family mealtime conflicts, work schedule interruptions, separate cooking requirements, and financial considerations. Facilitating factors were evaluated through questions regarding external fitness improvements, mood enhancement, bowel habit changes, and concentration levels. The questionnaire also included items about dietary sustainability, festive season challenges, travel-related adherence difficulties, and professional dietary consultation history, ensuring a comprehensive capture of all variables pertinent to understanding dietary modification patterns and their associated outcomes.

Data analysis

The data analysis was performed using IBM SPSS Statistics for Windows, Version 26.0 (released 2019; IBM Corp., Armonk, New York, United States). Descriptive statistics were computed for all variables, with continuous data presented as means and standard deviations and categorical data as frequencies and percentages. Chi-square tests were employed for categorical variables, with Fisher's exact test applied when more than 20% of the expected cell counts are less than five. Independent t-tests were used to compare continuous variables between intermittent fasting and alternative diet groups. A two-tailed p-value of <0.05 was considered statistically significant for all analyses. The analysis focused on comparing outcomes between intermittent fasting and alternative dietary approaches, examining barriers and facilitators, and assessing intervention effectiveness.

## Results

Table 1 shows the demographic and anthropometric characteristics of 432 participants seeking weight loss interventions. The mean age of participants was 27.93 ± 9.59 years, reflecting a predominantly young adult population. Gender distribution showed a slight female predominance, with 231 (53.4%) female participants compared to 196 (45.4%) male participants, and 5 (1.2%) didn’t reveal their gender. The geographical distribution indicated a substantial urban representation, with 295 (68.3%) participants from urban areas while 137 (31.7%) participants originated from rural settings. Educational stratification revealed that 383 (88.6%) participants were college-going students and completed graduate-level education. Forty-four completed higher secondary education (10.2%), while only 5 (1.2%) reported middle school as their highest educational qualification. Occupational distribution showed significant heterogeneity, with 190 students making up the largest segment (44%), followed by 150 salaried employees (34.7%) and 680 unemployed individuals (15.6%). Anthropometric measurements indicated a mean weight of 68.13 ± 13.76 kg and a mean height of 164.13 ± 9.79 cm. The calculated Body Mass Index (BMI) averaged 25.21 ± 4.16 kg/m^2^, suggesting that the study population, on average, fell within the overweight category according to World Health Organization classification criteria.

**Table 1:** Demographic and anthropometric characteristics of study participants (N = 432). *Mean ± Standard deviation.

Characteristics	Frequency (%)
Age in years	27.93 ± 9.59*
Gender	
Male	196 (45.4)
Female	231 (53.4)
Prefer not to say	5(1.2)
Residence	
Rural	137 (31.7)
Urban	295 (68.3)
Education	
Graduates	383 (88.6)
Higher secondary education	44 (10.2)
Middle school education	5 (1.2)
Occupation	
Agriculture	2 (0.5)
Business	18 (4.2)
Daily wager	2 (0.5)
Retired	2 (0.5)
Salaried	150 (34.7)
Student	190 (44)
Unemployed	68 (15.6)
Weight in kg	68.13 ± 13.76*
Height in cm	164.13 ± 9.79*
BMI in kg/m^2^	25.21 ± 4.16*

Table 2 shows the distribution of healthcare interventions and lifestyle modifications among study participants. Diet modifications were implemented by 143 (33.1%) participants, while 289 (66.9%) did not engage in healthcare interventions. Further stratification of dietary modifications demonstrated that 102 (23.6%) participants specifically adhered to intermittent fasting protocols, while 41 (9.5%) adopted alternative dietary approaches, including Paleo and ketogenic dietary regimens. Physical activity emerged as the predominant lifestyle modification, with 285 (66.0%) participants engaging in regular exercise routines, while 147 (34.0%) reported no structured physical activity. Mind-body interventions, specifically yoga and meditation, were practiced by 100 (23.1%) participants, with 332 (76.9%) not incorporating these practices into their lifestyle modifications. Pharmacological techniques and recreational activities demonstrated relatively lower adoption rates, with 80 (18.5%) participants utilizing these approaches, while 352 (81.5%) abstained from such interventions.

**Table 2:** Distribution of healthcare interventions and lifestyle modifications among study participants.

Different lifestyle modifications	Yes	No
Diet modifications	143	289
Physical activity	285	147
Yoga and meditation	100	332
Pharmacological techniques and recreational activities	80	352

In the study, among 143 subjects following any diet modifications, 5.21 (8.13) months was the mean (SD) duration of diet modifications, with a minimum of half a month to a maximum of 60 months. Table 3 shows the various characteristics of subjects following intermittent fasting and other diets. In this study, occupation is categorized as unemployed (nil and student), stable (salaried and retired with pensions), or unstable (agriculture, business, and daily wagers).

**Table 3:** Sociodemographic and anthropometric characteristics of individuals following intermittent fasting versus other dietary approaches. *Represented in mean ± standard deviation and an independent T test was used. #: Fisher’s exact test was used. !: Chi-square test was used Statistically significant when the p-value was less than 0.05.

Characteristics	Intermittent fasting (n = 102)	Other diets (n = 41)	p-value
Age in years*	27.09 ± 7.16	37.37 ± 13.07	0.001
Gender			
Female	55 (73.3%)	20 (26.7%)	0.085^#^
Male	45 (69.2%)	20 (30.8%)	
Prefer not to say	2 (66.7%)	1 (33.3%)	
Residence			
Rural	31 (72.1%)	12 (27.9%)	0.89^!^
Urban	71 (71%)	29 (29%)	
Education			
Graduates/postgraduates	94 (72.3%)	36 (27.7%)	0.42^#^
Middle school	8 (61.5%)	5 (38.5%)	
Occupation			
Unemployed	56 (80%)	14 (20%)	0.04^#^
Unstable occupation	3 (50%)	3 (50%)	
Stable occupation	43 (64.2%)	24 (35.8%)	
Height in cm*	164.51 ± 10.30	162.73 ± 11.16	0.53
Weight in kg*	71.98 ± 14.73	71.27 ± 15.72	0.49
BMI*	26.43 ± 3.74	26.69 ± 4.14	0.58

Analysis revealed a significant age disparity between the groups, with intermittent fasting practitioners displaying a mean age of 27.09 years (SD = 7.16) compared to 37.37 years (SD = 13.07) among those following other diets (p = 0.001). Gender distribution analysis showed a predominance of females in both groups, with 55 participants (73.3%) in the intermittent fasting group and 20 participants (26.7%) in the other diet group. Male participation comprised 45 individuals (69.2%) in the intermittent fasting cohort and 20 (30.8%) in the alternative diets group. A minimal number of participants (n = 3) preferred not to disclose their gender, with 2 (66.7%) in the intermittent fasting group and 1 (33.3%) in the other diets group.

Regarding residential distribution, urban residents constituted the majority, with 71 participants (71%) following intermittent fasting and 29 (29%) pursuing other diets. Rural participation showed similar proportions, with 31 (72.1%) and 12 (27.9%) participants in respective groups. Educational status indicated a predominance of graduates/postgraduates (n = 130), with 94 (72.3%) practicing intermittent fasting and 36 (27.7%) following other diets. Occupational status demonstrated statistically significant differences (p = 0.04), with unemployed individuals showing higher participation in intermittent fasting (n = 56, 80%) compared to other diets (n = 14, 20%). Those with stable occupations were distributed as 43 (64.2%) in intermittent fasting and 24 (35.8%) in other diets, while unstable occupation categories showed equal distribution (n = 3, 50% in each group).

Anthropometric measurements revealed comparable characteristics between groups. Mean height was 164.51 cm (SD = 10.30) for intermittent fasting practitioners versus 162.73 cm (SD = 11.16) for other diets (p = 0.53). The mean weight was 71.98 kg (SD = 14.73) and 71.27 kg (SD = 15.72), respectively (p = 0.49). Body Mass Index (BMI) measurements were similar, with means of 26.43 (SD = 3.74) for intermittent fasting and 26.69 (SD = 4.14) for other diets (p = 0.58).

Table 4 shows the comparative analysis of adherence patterns, outcomes, and challenges between intermittent fasting and alternative dietary interventions. Regular adherence demonstrated statistically significant differences between the groups (p = 0.03), with intermittent fasting participants showing higher compliance rates (n = 67, 65.7%) compared to alternative diet adherents (n = 17, 41.5%). Regarding systemic side effects, 34 participants (33.3%) in the intermittent fasting group and 10 (24.4%) in the alternative diet group reported adverse effects, though this difference was not statistically significant (p = 0.53).

**Table 4:** Comparative analysis of adherence patterns, outcomes, and challenges between intermittent fasting and alternative dietary interventions. *: Fischer’s exact test. #: Chi-square test was used. The association was computed with the outcome as agree, disagree, or neutral for each response between intermittent fasting and other diets, and the difference was considered statistically significant when the p-value was less than 0.05.

Characteristics	Intermittent fasting (n = 102)	Other diet (n = 41)	p-value
Regular follower	67 (65.7%)	17 (41.5%)	0.03*
Systemic side effects	34 (33.3%)	10 (24.4%)	0.53^#^
Significant weight loss	73(71.6%)	31 (75.6%)	0.74*
Mood changes and memory problems	34 (33.3%)	14 (34.1%)	0.75^#^
Affect sleep duration	37 (36.3%)	10 (24.4%)	0.38^#^
Gained weight while defaulting	69 (67.6%)	23 (56.1%)	0.34*
Easy to follow	59 (57.8%)	19 (46.3%)	0.46^#^
Infrequent during the festive season	75 (73.5%)	29 (70.7%)	0.44*
Infrequent during travel	84 (82.4%)	32 (78%)	0.8*
Consulted a dietician beforehand	19 (18.6%)	10 (24.4%)	0.71^#^

Weight loss efficacy was comparable between groups, with 73 participants (71.6%) in the intermittent fasting cohort and 31 (75.6%) in the alternative diet group reporting significant weight reduction (p = 0.74). Psychological impacts, specifically mood alterations and memory-related concerns, were reported by similar proportions: 34 (33.3%) in the intermittent fasting group and 14 (34.1%) in the alternative diet group (p = 0.75). Sleep duration effects were noted by 37 participants (36.3%) following intermittent fasting compared to 10 (24.4%) in the alternative diet group (p = 0.38). Weight regains during dietary non-adherence were reported by 69 participants (67.6%) in the intermittent fasting group and 23 (56.1%) in the alternative diet group (p = 0.34).

Regarding dietary sustainability, 59 participants (57.8%) in the intermittent fasting group and 19 (46.3%) in the alternative diet group reported their respective approaches as easily manageable (p = 0.46). Adherence challenges were particularly evident during festive seasons, affecting 75 participants (73.5%) in the intermittent fasting group and 29 (70.7%) in the alternative diet group (p = 0.44). Similarly, travel-related adherence difficulties were reported by 84 participants (82.4%) practicing intermittent fasting and 32 (78%) following alternative diets (p = 0.8). Professional dietary consultation prior to intervention initiation was notably low across both groups, with only 19 participants (18.6%) from the intermittent fasting group and 10 (24.4%) from the alternative diet group seeking dietician guidance (p = 0.71).

Table 5 shows the comparative analysis of barriers and facilitators between intermittent fasting and alternative dietary interventions. Among the barriers identified, hunger at bizarre timings was reported as the most prevalent challenge in both groups, affecting 55 (53.9%) intermittent fasting participants and 22 (53.7%) subjects following other diets. Timing-related constraints were notably significant, with 50 (49%) intermittent fasting participants and 20 (48.8%) subjects on other diets reporting difficulties with diet timing. Family mealtime conflicts posed substantial challenges, affecting 43 (42.2%) and 21 (51.2%) participants in the intermittent fasting and other diet groups, respectively.

**Table 5:** Comparative analysis of barriers and facilitators between intermittent fasting and alternative dietary interventions. *: Fischer’s exact test. #: Chi-square test was used. The association was computed with the outcome as agree, disagree, or neutral for each response between intermittent fasting and other diets, and the difference was considered statistically significant when the p-value was less than 0.05.

Barriers and facilitators	Intermittent fasting (n = 102)	Other diet (n = 41)	p-value
Barriers			
Timing of the diet	50 (49%)	20 (48.8%)	0.74^#^
Issue of separate cooking	42 (41.2%)	20 (48.8%)	0.45^#^
Nil immediate effect on weight	26 (25.5%)	13 (31.7%)	0.39^#^
Affect daily routine	31 (30.4%)	13 (31.7%)	0.86^#^
Affects sleep quality	21 (20.6%)	8 (19.5%)	0.79^#^
Expensive	28 (27.5%)	16 (39%)	0.26^#^
Family mealtime conflict	43 (42.2%)	21 (51.2%)	0.52^#^
Hungry at bizarre timings	55 (53.9%)	22 (53.7%)	0.23^#^
Work schedule conflict	39 (38.2%)	17 (41.5%)	0.31^#^
Facilitators			
Externally fit	75 (73.5%)	31 (75.6%)	0.93*
Improves concentration	71 (69.6%)	27 (65.9%)	0.19*
Improved bowel habits	72 (70.6%)	26 (63.4%)	0.47*
Improved skin texture	63 (61.8%)	22 (53.7%)	0.66*
Throughout active	70 (68.6%)	27 (65.9%)	0.85*
Improved mood	73 (71.6%)	23 (56.1%)	0.21*

Regarding work-related challenges, work schedule conflicts were reported by 39 (38.2%) participants in the intermittent fasting group and 17 (41.5%) in the other diet group. The issue of separate cooking emerged as a considerable barrier, affecting 42 (41.2%) intermittent fasting participants and 20 (48.8%) subjects following other diets. Financial considerations were more prominent in the other diet group, with 16 (39%) participants reporting expenses as a barrier compared to 28 (27.5%) in the intermittent fasting group.

In terms of facilitators, external fitness was the most commonly reported benefit across both groups, with 75 (73.5%) intermittent fasting participants and 31 (75.6%) subjects on other diets noting this advantage. Improved mood was reported by 73 (71.6%) participants in the intermittent fasting group, compared to 23 (56.1%) in the other diet group. Enhanced bowel habits were noted by 72 (70.6%) intermittent fasting participants and 26 (63.4%) subjects following other diets. Improved concentration was reported by 71 (69.6%) and 27 (65.9%) participants in the respective groups. Notably, no statistically significant differences (p > 0.05) were observed between the two groups for any of the barriers or facilitators studied, suggesting comparable experiences across both dietary approaches.

## Discussion

The present study investigated dietary modification patterns among individuals attempting weight loss in Tamilnadu, South India, with a comprehensive exploration of interventions, dietary practices, and associated barriers and facilitators. Our findings reveal intricate dynamics of dietary modifications, particularly highlighting the prevalence and characteristics of intermittent fasting and alternative dietary approaches.

Age-Related Dietary Intervention Patterns

The study demonstrated a significant age-related distinction in dietary intervention selection. Intermittent fasting emerged predominantly among younger participants, with a mean age of 27.09 years, compared to alternative dietary approaches, with a mean age of 37.37 years. This observation aligns with Fanti et al. (2021), who suggested that younger populations exhibit greater dietary experimentation and technological adaptability in weight management strategies [7]. The age-related variation might be attributed to increased digital health literacy, social media influence, and contemporary wellness trends among younger demographics.

Demographic Characteristics and Dietary Modifications

Despite minimal statistically significant variations across gender, residence, and educational backgrounds, our findings reveal nuanced occupational influences. Unemployed individuals demonstrated a higher propensity for intermittent fasting (56, 80%), suggesting potential correlations between employment status and dietary intervention accessibility. This observation resonates with Helland and Nordbotten's (2021) mixed-methods research, which highlighted socioeconomic factors' role in dietary modifications [26].

Effectiveness and Physiological Implications

Participants practicing intermittent fasting reported promising weight loss outcomes, with 73 (71.6%) experiencing significant weight reduction. These results corroborate Varady et al.'s (2022) clinical review, which emphasized intermittent fasting's potential for weight management [27]. However, our study also revealed heightened vulnerability to regain weight during non-adherence, underscoring the importance of sustainable dietary strategies.

Cognitive and Psychological Dimensions

Remarkably, intermittent fasting participants reported substantial cognitive and psychological benefits. In this study, 73 (71.6%) experienced mood improvements, compared to 23 (56.1%) in alternative dietary groups. Concentration enhancements were observed in 69.6% of intermittent fasting participants, supporting emerging research on nutritional interventions' neurological impacts [28].

Barriers and Facilitators

The study comprehensively mapped dietary intervention challenges. Timing emerged as a critical barrier, affecting nearly 50% of participants across both groups. Lifestyle disruptions, including family mealtime and work schedule conflicts, presented significant impediments. These findings resonate with Abel et al.'s (2018) qualitative exploration of dietary change barriers, emphasizing the complex interplay between personal, social, and occupational factors [17].

Physiological and Holistic Health Outcomes

Beyond weight management, participants reported multifaceted health improvements. Intermittent fasting demonstrated notable benefits in bowel habits (72, 70.6%), skin texture (63, 61.8%), and sustained activity levels (70, 68.8%). These observations align with Santos and Macedo's (2018) research on intermittent fasting's comprehensive metabolic impacts [23].

Clinical Significance

The study's clinical significance lies in its nuanced elucidation of dietary modification patterns among young Indian adults. The research provides critical insights for healthcare practitioners and nutritional professionals by systematically examining intermittent fasting and alternative dietary approaches. The findings highlight the necessity of personalized, context-sensitive nutritional interventions that consider individual demographic, occupational, and physiological characteristics.

The observed age-related variations in dietary intervention selection underscore the importance of tailored nutritional counseling. Healthcare providers can leverage these insights to develop targeted strategies addressing specific demographic needs. The study reveals that younger populations demonstrate greater dietary experimentation, suggesting an opportunity for proactive nutritional education and evidence-based guidance.

Moreover, the comprehensive mapping of barriers and facilitators offers invaluable clinical perspectives. Understanding individuals' multifaceted challenges during dietary modifications enables more empathetic and effective intervention design. The research illuminates the intricate interactions between lifestyle factors, psychological well-being, and nutritional choices.

The cognitive and psychological benefits associated with intermittent fasting warrant further clinical investigation. The observed improvements in mood, concentration, and overall well-being suggest potential broader implications beyond weight management. Clinicians can consider these holistic outcomes when recommending dietary interventions, emphasizing comprehensive health optimization.

The study also highlights the complex relationship between employment status and dietary modifications, providing critical insights for socioeconomic-sensitive nutritional strategies. By recognizing the unique challenges faced by different occupational groups, healthcare professionals can develop more inclusive and accessible dietary intervention protocols.

Strength of the study

The study's primary strength lies in its comprehensive and methodologically rigorous approach to exploring dietary modification patterns. The robust sample size of 432 participants provides statistically significant insights into weight loss interventions. The meticulous demographic stratification and multidimensional analysis offer unprecedented depth in understanding dietary intervention dynamics. The research's innovative approach of comparing intermittent fasting with alternative dietary strategies enables nuanced comparative insights. The study transcends traditional weight loss research paradigms by systematically examining barriers, facilitators, and physiological and psychological outcomes. The study's geographical focus on Tamilnadu, South India, addresses a critical research gap in regional dietary modification patterns. The inclusion of both quantitative measurements and qualitative insights provides a holistic research perspective.

Limitations

The predominantly urban, highly educated sample might limit generalizability across diverse socioeconomic contexts. The self-reported nature of dietary and health outcomes introduces potential recall and reporting biases. The cross-sectional study design prevents establishing definitive causal relationships between dietary interventions and observed outcomes. Long-term physiological and psychological implications remain unexplored, requiring longitudinal research for a more comprehensive understanding. The study's reliance on participant self-reporting may introduce a subjective interpretation of dietary adherence and health improvements. The multifaceted nature of weight loss is driven by individuals' motivational factors, which manifest in the concurrent adoption of various interventions, including dietary modifications, physical activity regimens, and lifestyle adjustments. These interrelated factors potentially function as confounders or effect modifiers when analyzing weight loss outcomes and their associated determinants, requiring careful consideration in intervention assessment and outcome interpretation. Objective biochemical and anthropometric measurements would enhance result validation. The geographical restriction to Tamilnadu limits broader regional or national applicability. Cultural, dietary, and lifestyle variations across different Indian states might yield divergent results. The study's relatively short mean duration of dietary modification (5.21 months) may not capture sustained long-term dietary intervention impacts. Extended observation periods could provide more nuanced insights into dietary modification sustainability.

Recommendations

Future research requires a methodologically sophisticated approach, prioritizing longitudinal investigations to elucidate sustained dietary intervention outcomes. Imperative strategies include implementing standardized biochemical assessments and objective health metrics to enhance epistemological reliability. Researchers must strategically diversify sampling methodologies, encompassing participants across heterogeneous socioeconomic spectrums, educational strata, and geographical demarcations to augment result generalizability. Healthcare practitioners should design algorithmically personalized nutritional intervention protocols that integrate comprehensive individual demographic, occupational, and psychological profiling. Interdisciplinary research paradigms integrating nutritional science, psychological frameworks, and neurobiological mechanisms can potentially unveil sophisticated insights into cognitive modulation associated with dietary interventions. Additionally, developing culturally nuanced, contextually adaptive dietary intervention guidelines that acknowledge regional dietary preferences and intricate lifestyle constraints would substantially optimize practical implementation and translational potential.

## Conclusions

The study provides unprecedented insights into dietary modification patterns among young Indian adults, highlighting intermittent fasting's potential as a dynamic weight management strategy. The research transcends traditional nutritional intervention paradigms by comprehensively exploring demographic, physiological, and psychological dimensions. The findings underscore the complex interplay between dietary choices, individual characteristics, and holistic health outcomes. While demonstrating promising weight loss and cognitive benefits, the study also reveals significant barriers and individual variability in dietary modification approaches. These insights emphasize the necessity of personalized, context-sensitive nutritional interventions. Future research should focus on longitudinal assessments, diverse population sampling, and interdisciplinary investigations to further elucidate dietary modification mechanisms and optimize intervention strategies.

## Appendices

Questionnaire

Demographic and Anthropometric Characteristics

1. Age in completed years

2. Gender: Male/female/prefer not to say

3. Type of residence: Urban/rural

4. Education status: No formal education/primary (up to fifth standard)/middle (sixth to eighth standard)/high (ninth and 10th standard)/higher secondary (11th and 12th standard)/graduate and above

5. Occupation: Agriculture/business/salaried/daily wager/retired/pension/unemployed/student

6. Height (in centimeter)

7. Weight (in kilograms)

Questions on Diet Practices

Type of practice for weight loss: Diet modifications/physical activity yoga and meditation/others

Special type of diet followed: Paleo diet/keto diet/intermittent fasting/others

How long (in months) are you following the diet modifications?

Table 6 shows the questions of characteristics of diet practices followed by study participants.

**Table 6:** Characteristics of diet practices.

Characteristics of diet practices	Strongly agree	Agree	Neutral	Disagree	Strongly disagree
I am a regular follower of diet practice					
I have experienced side effects like gastritis, headache and change of body odour during the time					
I have achieved weight loss with the practice					
I have gained weight while leaving the practice					
I am experiencing mood changes and memory problems after the start of practice					
The diet is affecting my sleep duration					
The diet is I am taking is an easy to follow at all times					
I am not able to follow the diet during festive season					
I am not able to follow the diet during trips					
I have consulted a dietician before the start of the diet					

Table 7 shows the questions of barriers of diet practices.

**Table 7:** Barriers of diet practices.

Barriers of diet practices	Strongly agree	Agree	Neutral	Disagree	Strongly disagree
The timing of the diet practice makes it difficult					
My diet has to be separately cooked					
The diet has no effect on my weight					
The diet alters your daily routine					
The diet practice affects my sleep quality					
The diet practice is expensive					
The diet practice is not in line with family meal schedule					
The dietary practice makes me hunger at bizarre timings					
The dietary practice makes me gain weight when I quit					
The diet practice is not in line with my work schedule					

Table 8 shows the questions of facilitators of diet practices.

**Table 8:** Facilitators of diet practices.

Facilitators of diet practices	Strongly agree	Agree	Neutral	Disagree	Strongly disagree
The diet practice made me fit externally					
The diet practice improves concentration					
The diet practice improved my bowel habits					
The diet practice improved my skin texture					
The diet practice affects my sleep quality					
The diet practice made me active					
The diet practice improved my mood					
